# Identifying enthesitis in the sacroiliac joints in patients with axial spondyloarthritis by readers of varying experience: impact of the learning progress

**DOI:** 10.1186/s41927-024-00397-4

**Published:** 2024-08-21

**Authors:** Dong Liu, Jiaoshi Zhao, Churong Lin, Budian Liu, Jinwei Li, Yuxuan Zhang, Ou Jin, Jieruo Gu

**Affiliations:** 1 Department of Rheumatology, the Third Affiliated Hospital of Sun Yat-Sen University, Guangzhou, China; 2 Department of Radiology, the Third Affiliated Hospital of Sun Yat-Sen University, Guangzhou, China

**Keywords:** MRI, Enthesitis, Spondyloarthritis, Sacroiliac joint, Experience and training

## Abstract

**Background:**

This study aimed to investigate the accuracy of identifying enthesitis along with other inflammatory lesions and structural lesions on the MRI of the sacroiliac joints (SIJ) by readers of varying experience and how training sessions and workshops could help improve the accuracy.

**Methods:**

A total of 224 patients with clinical diagnosis of axial spondyloarthritis who underwent SIJ MRI examinations were retrospectively included in this study. Three readers with 5 years, 3 years and 1 year of experience in musculoskeletal imaging were invited to review the SIJ MRI images independently, while the imaging reports of a senior radiologist (> 10 years’ experience) were used as reference. After the first round of image review, a training session and a workshop on the imaging of SIJ in spondyloarthritis were held and the three readers were asked to review the images in the second round. We calculated the accuracy of identifying inflammatory and structural lesions of the three readers as well as the intra-reader agreement.

**Results:**

Enthesitis could be observed in 52.23% of the axial spondyloarthritis patients, while 81.58% of the patients with enthesitis were accompanied with bone marrow edema. All the three readers showed better accuracy at identifying structural lesions than inflammatory lesions. In the first round of image review, the three readers only correctly identified 15.07%, 2.94% and 0.74% of the enthesitis sites. After the training session and workshop, the accuracy rose to 61.03%, 39.34% and 20.22%. The intra-reader agreement of enthesitis calculated as Cohen’s kappa was 0.23, 0.034 and 0.014, respectively.

**Conclusion:**

Readers with less experience in musculoskeletal imaging showed lower accuracy of identifying inflammatory lesions, notably enthesitis. Training sessions and workshops could help improve the diagnostic accuracy of the junior readers.

## Introduction

Spondyloarthritis (SpA) belongs in the group of diseases known as inflammatory arthropathies, and could be further classified into axial spondyloarthritis (axSpA) and peripheral spondyloarthritis (pSpA) depending on whether the patients exhibit axial manifestations or only peripheral manifestations [1, 2]. Either in axSpA or pSpA, enthesitis is considered the hallmark of the SpA disease entity [3, 4]. Enthesis refers to the anatomic interface between tendons, ligaments, capsules, fascia and bones, while the concept of “enthesis organ” denotes the anatomic structure of enthesis in conjunction with the adjacent structures such as bursae, periosteal fibrocartilage and the synovial-covered fat pads. Inflammation at the such insertional sites is considered as enthesitis [5, 6]. 

According to the SpondyloArthritis international Society (ASAS) classification criteria for axSpA and pSpA [3, 4], enthesitis is included in the set of SpA features, and findings of enthesitis could facilitate the early diagnosis of SpA, which is increasingly relevant since timely institution of biologics could effectively relieve the inflammatory symptoms and slow the radiographic progression [7]. As per the definition of enthesitis by the ASAS classification criteria, only past or present spontaneous pain or tenderness during physical examination at the insertion site of the Achilles tendon or plantar fascia could serve as a SpA feature [3]. However, technological advances in ultrasound [7] and magnetic resonance imaging (MRI) [8] provided clinicians with more imaging options to properly identify enthesitis in patients with SpA. Ultrasound is often applied to visualize enthesitis at the extremities [9], but its capacity of visualizing the deep-seated entheses is limited, especially the entheses inserting into vertebrae and the pelvic entheses [10]. Compared with physical examination and ultrasound, MRI could visualize enthesitis with excellent spatial resolution and significantly higher diagnostic sensitivity [11, 12]. 

The sacroiliac joint (SIJ) MRI could enable the clear visualization of both inflammatory lesions and structural changes in the SIJ and provide direct proof of axial inflammation, hence the inclusion in the imaging arm of the ASAS classification criteria [3]. However, as is stipulated by the ASAS classification criteria, only bone marrow edema (BME) located in a typical anatomical area (subchondral bone) could comprise a positive SIJ MRI [13]. Other inflammatory pathologies, including enthesitis and capsulitis, and structural lesions such as erosions, are not included in the definition of a positive SIJ MRI. Another interesting observation is that enthesitis is often overlooked in the image review in clinical practice, especially by the less experienced readers.

This study intends to investigate the accuracy of identifying enthesitis along with other inflammatory lesions and structural lesions including BME, erosions, fatty lesions, and ankylosis on the MRI of the sacroiliac joints in axSpA patients by readers with varying experience, and whether training sessions and workshops could help junior readers increase their accuracy in identifying enthesitis. This study also intends to investigate the distribution of enthesitis in different locations of the SIJ MRI in patients with axSpA. This study highlights the importance of experience and training in the interpretation of musculoskeletal imaging, and suggests that training sessions and workshops could help junior readers achieve better accuracy during image review.

## Methods

### Design

This is an accuracy study investigating the accuracy of identifying enthesitis along with other inflammatory lesions and structural lesions in the SIJ MRI of axSpA patients by readers with varying experience. This study was approved by the Ethical Committee of the hospital.

### Study population

Patients receiving clinical diagnosis of axSpA were considered eligible for this imaging study, and the SIJ MRI images were retrospectively retrieved. These participants visited the rheumatology clinic of the Third Affiliated Hospital of Sun Yat-sen University from January 2018 to May 2022 with complaints of axial or peripheral symptoms, and were referred for SIJ MRI with clinical suspicion of sacroiliitis. The inclusion criteria were the fulfillment of the ASAS classification criteria for axSpA [3]. Exclusion criteria consisted of age below 18 years, concomitant malignancies, pregnancy and contraindications for MRI.

### MRI protocol

All patients underwent MRI scanning of the sacroiliac joints in the supine position using 3.0 T MR scanner (SignaTM Architect, GE Healthcare, Milwaukee, WI) and a 3.0 T MRI scanner (Magnetom Prisma, Siemens Healthineers, Erlangen, Germany) and a 3.0T MR scanner (MR750; GE Healthcare, Milwaukee, WI, USA). The routine SIJ MRI examination consisted of T2-weighted fat-suppressed turbo spin echo (T2-FS) sequence, T1-weighted images (T1WI) sequence, T1-weighted images with fat saturation (T1-FS) sequence in a semi-coronal orientation and T2-FS sequences in a semi-axial orientation for the SIJ were available. The scanning parameters could be seen in Table 1.

**Table 1:** Imaging parameters of MRI sequences

Parameters	T1WI	T1-FS	T2-FS	T2-FS
Plain	Coronal	Coronal	Coronal	Transverse
No.of section	18	18	18	18
Section thickness (mm)	3	3	3	3
Section gap (mm)	0.6	0.6	0.6	0.6
Field of view (mm)	240*240	240*240	240*240	280*280
Voxel size (mm)	0.8*0.9*3	0.9*0.9*3	0.8*0.9*3	0.8*1.0*3
Repetition time (msec)	624	725	4304	4561
Echo time (msec)	7	7	68	68
Acquisition time	1:30	1:54	2:05	1:36

### Image review

The SIJ MRI scans were first reviewed by an expert radiologist with over 10 years of experience in musculoskeletal imaging, blinded to the patient information and other laboratory and imaging findings. The inflammatory lesions and the structural changes were identified based on the 2019 updated definitions of SIJ MRI lesions [13].

1. BME: a hyperintense signal on water-sensitive images (STIR or T2-FS), which is clearly present and located in a typical anatomical area (subchondral bone).
2. Enthesitis: Increased signal in bone marrow and/or soft tissue on STIR or T2-FS images at sites where ligaments and tendons attach to bone, but not including the inter-osseous ligaments of the sacroiliac joint.
3. Erosion: A step-down contour in subchondral bone associated with full-thickness loss of the dark appearance of the subchondral cortex on T1-W images.
4. Fat lesion: Bright signal seen on T1-W images that is brighter than normal bone marrow.
5. Ankylosis: Abnormal bright signal on a T1-W images that bridges the joint so that there is continuity of bone marrow signal between the ilium and sacrum.

**Fig. 1:**
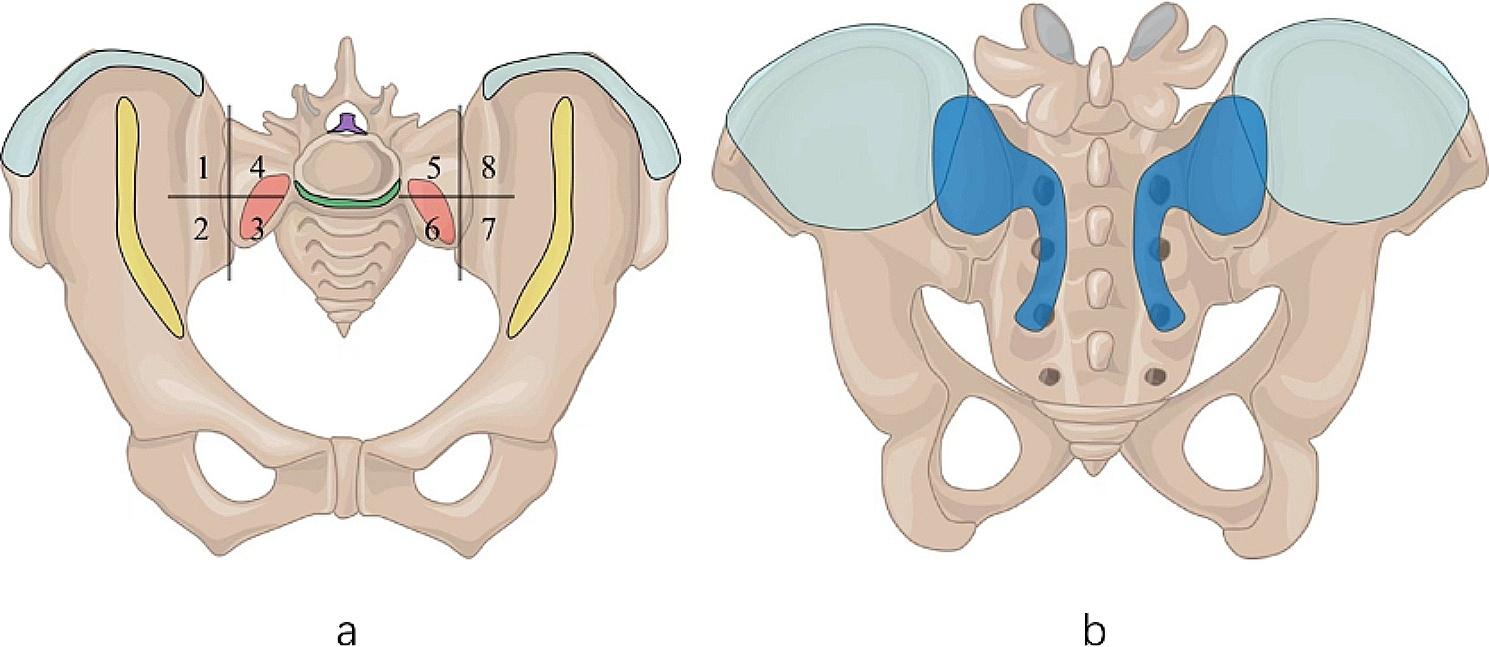
Sites of enthesitis around the sacroiliac joint. (**a**) Anterior sacroiliac ligament insertion (red and yellow), longitudinal ligament insertion (green and purple), iliac crest and wing (blue). (**b**) Posterior sacroiliac ligament insertion (dark blue), iliac crest and wing (blue)

This reader was asked to record the locations of these imaging findings. The following locations were examined for presence of enthesitis: (1) ligaments of the sacroiliac joint, except the interosseous ligament, recorded in the form of eight quadrants.; (2) the longitudinal ligaments of the visualized lumbar vertebrae ; (3) the iliac crest and wing. (Fig. 1) Enthesitis of the pubic symphysis was not evaluated since the FOV adopted in this study could not effectively cover this area. MRI interpretations of this reader were established as the reference for the following reading exercises.

After the reference has been established, 3 readers were invited to review the SIJ MRI scans. Reader 1, 2 and 3 each has 5 years, 3 years and 1 year of experience in musculoskeletal imaging. In order to simulate the clinical scenario in which the lesions often considered “less important” were often overlooked, such as enthesitis, these readers were blinded to the objective of this study, as well as the clinical information and other imaging results. All three readers were required to complete two rounds of imaging review, with an interval of one month between the two reading exercises. Both inflammatory lesions and structural lesions were scored on a binary scale in different locations of the SIJ MRI.

After finishing the first round, the objective of this study was revealed to the readers, and a training session and a workshop was held to instruct the readers how to properly identify enthesitis in the SIJ MRI. Specifically, the three junior readers were asked to attend a lecture given by the senior radiologist on the imaging manifestations of axSpA on SIJ MRI, with an emphasis on enthesitis. The training session was followed by an interactive workshop, during which the three readers were asked to interpret 20 SIJ MRI scans not belonging in the previous reading exercise, and the senior radiologist gave feedback to the three readers explaining what to look for when reviewing the SIJ MRI images. One month after the first reading exercise, the three readers were asked to review these SIJ MRI scans again and report the locations of the imaging findings.

### Statistical analysis

Statistical analysis was carried out on the R platform (version 4.2.0). Characteristics of the study participants were presented with standard descriptive statistics. Based on the reference established by the expert radiologist, the prevalences of enthesitis, BME, erosions, fat lesions and ankylosis were calculated. Accuracy of identifying enthesitis, BME, erosions, fat lesions and ankylosis by the three readers of varying experience were calculated using the reports of the expert radiologist as the reference. Cohen’s kappa (k) was calculated to evaluate the intra-reader agreement. A *p*-value less than 0.05 is considered statistically significant.

## Results

### Demographic characteristics

Two hundred and twenty-four patients diagnosed as axSpA were included in this study, with an average age of 31.83 ± 9.84 years. Among the study subjects, 158 (70.54%) were male and 66 (29.46%) were female. 93.72% of the participants were HLA-B27 positive, while 70.98% of the participants presented radiological sacroiliitis. The median disease duration of study subjects was 3.9 years (interquartile range 0.67-5.0). The descriptive statistics were summarized in Table 2.

**Table 2:** Characteristics of the 224 study participants

Characteristics	Total patients (*n* = 224)
Sex ‡	
Male (%)	70.54
Female (%)	29.56
Age (years)	31.83 ± 9.84
Disease duration (years)	3.9[0.67-5.0]
CRP (mg/L)	4.05 [1.4–13.7]
ESR (mm/h)	27.5 [16.75–52.25]
HLA-B27 positivity (%) ‡	93.72
Radiological sacroiliitis (%) ‡	70.98

Note.—For data that fulfilled normal distribution, data are presented as means ± standard deviation; for data that did not fulfill normal distribution, data are presented as median [interquartile range]

‡ Data are percentages

### Prevalences of inflammatory lesions and structural lesions on SIJ MRI

Enthesitis was seen in 52.23% of the axSpA patients, with an average of 1.26 sites of enthesitis in each patient. BME could be seen in 56.17% of the patients with an average of 2.27 sites. For structural lesions, erosions and fat lesions could be seen in 83.04% and 68.30% of the patients, with an average of 4.91 sites and 3.38 sites. Ankylosis was seen in 16.96% of the axSpA patients.

We also analyzed the association between enthesitis and other imaging findings. We found that 81.58% of the patients with enthesitis were also accompanied with BME, while erosions, fat lesions and ankylosis could be seen in 92.11%, 73.68% and 19.30% of the patients with enthesitis. Conversely, in 18.42% of the patients, enthesitis occurred independent of BME.

### Distribution of enthesitis on SIJ MRI

**Fig. 2:**
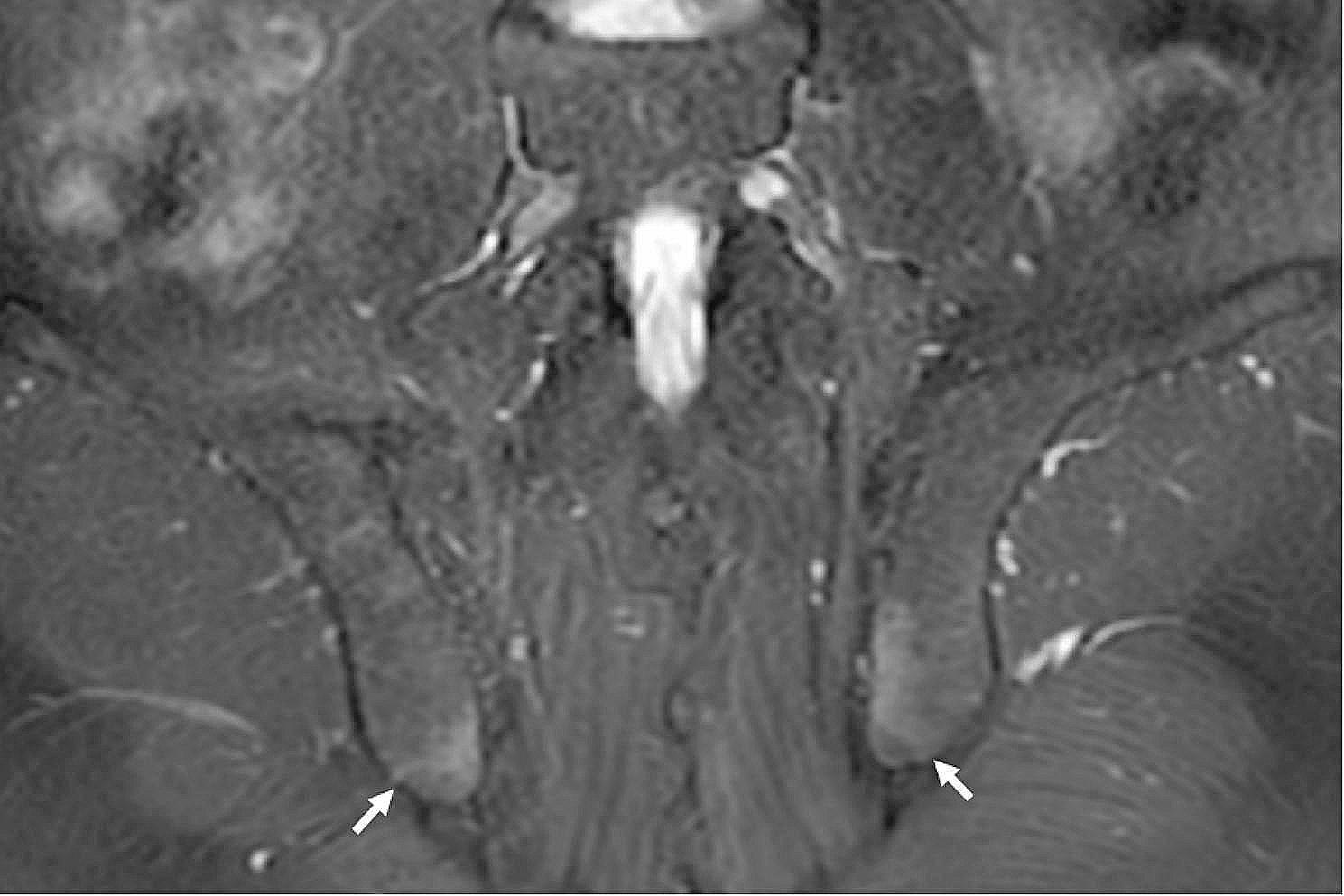
Enthesitis in a 37-year-old male with axial spondyloarthritis. Coronal oblique T2-FS MR images exhibited bilateral posterior sacroiliac ligament enthesitis at the inferior iliac quadrant (arrow)

**Fig. 3:**
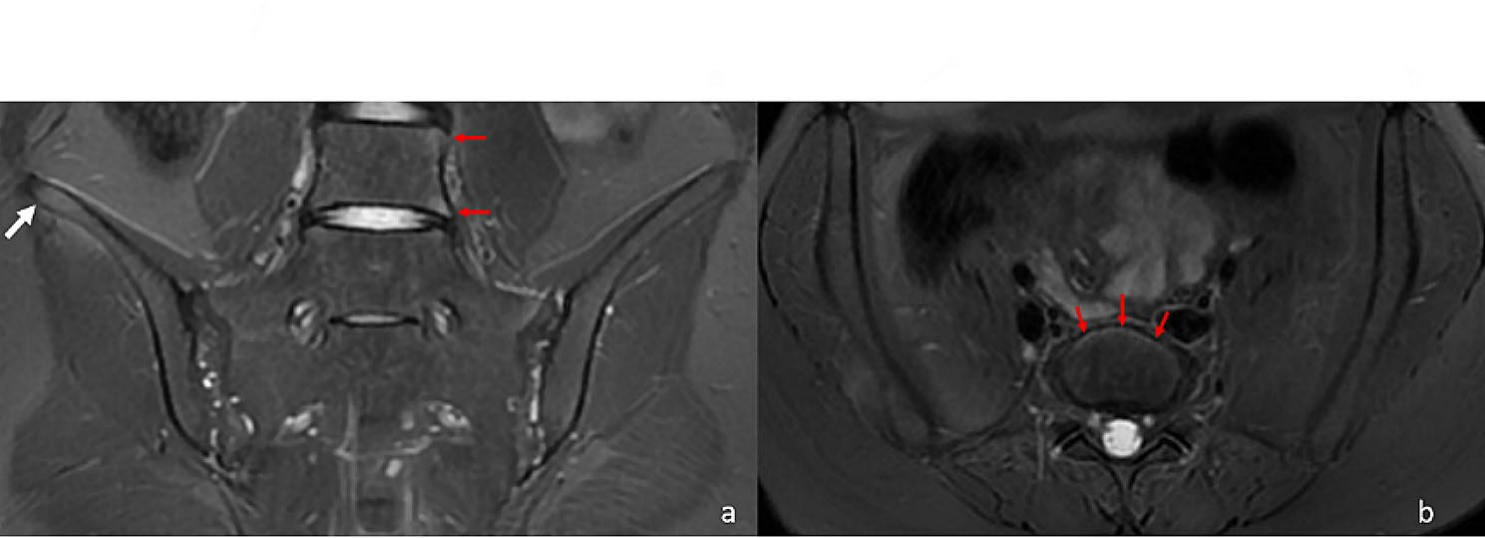
Enthesitis in a 24-year-old female with axial spondyloarthritis. (**a**) Coronal oblique T2-FS MR image exhibited enthesitis at the right iliac crest and wing, visualized as bone marrow edema and hyperintense signal at the adjacent ligaments (white arrow), as well as anterior longitudinal ligament enthesitis at the L5 vertebra (red arrow). (**b**) Transverse T2-FS MR image confirmed the presence of anterior longitudinal ligament enthesitis at the L5 vertebra (red arrow)

**Fig. 4:**
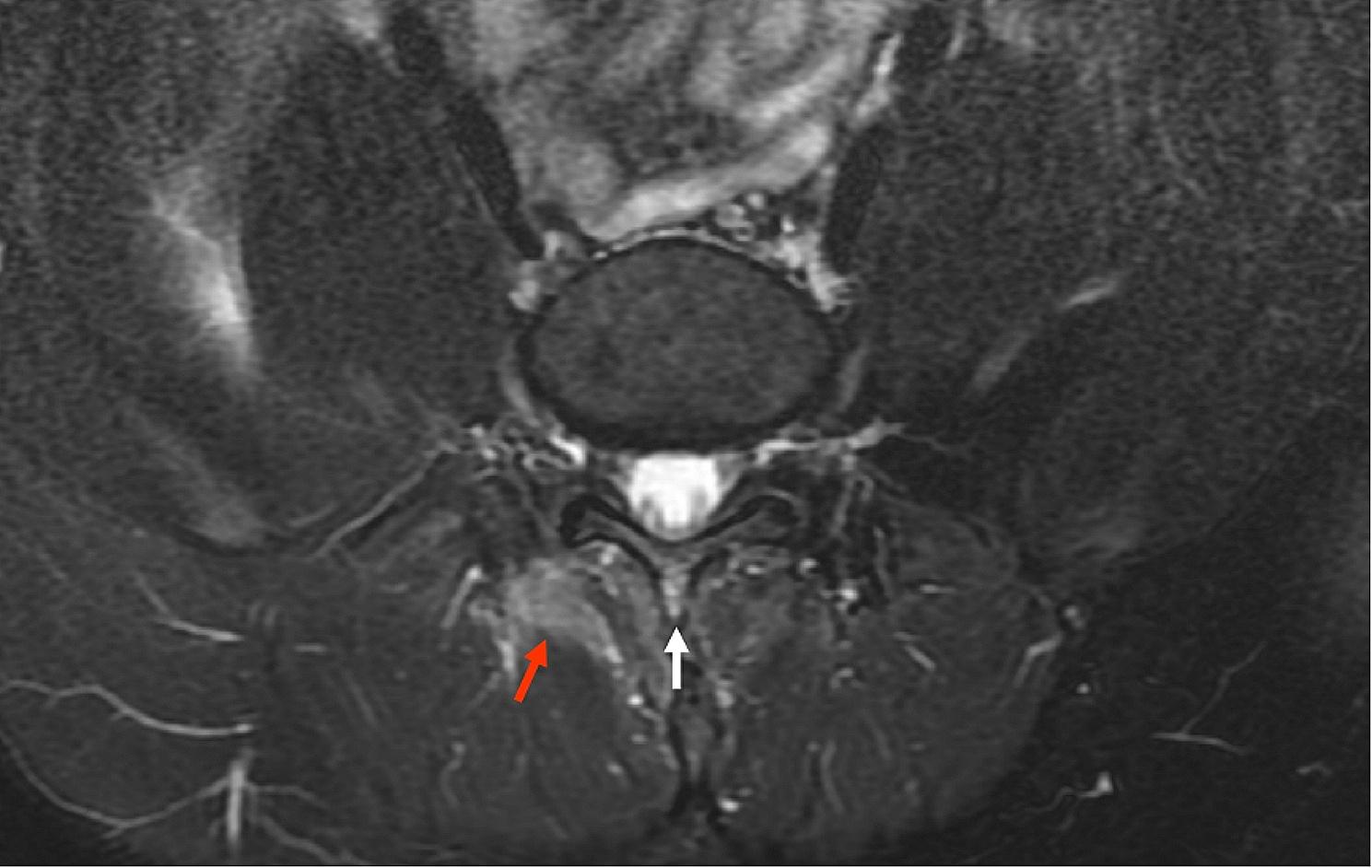
Enthesitis in a 19-year-old male with axial spondyloarthritis. Transverse MR image exhibited enthesitis at the spinous process of the L5 vertebra, visualized as bone marrow edema at the spinous process (white arrow) and inflammation at the adjacent soft tissue (red arrow)

The distribution of the enthesitis in different locations on SIJ MRI could be seen in Table 3. The most commonly affected entheses were the posterior sacroiliac ligament entheses attaching to the left lower iliac quadrant (21.43%) and right lower iliac quadrant (20.54%). (Fig. 2) Enthesitis was also common in the left lower sacral quadrant (16.52%) and right lower sacral quadrant (15.18%). Enthesitis at the left and right iliac crest/wing could be seen in 7.59% and 8.48% of the patients. (Fig. 3a) Enthesitis was sometimes seen in the lumbar longitudinal ligament entheses (4.91%). (Figs 3 and 4)

**Table 3:** Distribution of enthesitis at different compartments around the sacroiliac joint in patients with axial spondyloarthritis

Location		Enthesitis Prevalence
Sacroiliac ligaments (Excluding interosseous ligament)	Left upper sacral quadrant	3.57%
	Left lower sacral quadrant	16.52%
	Left upper iliac quadrant	8.04%
	Left lower iliac quadrant	21.43%
	Right upper sacral quadrant	4.46%
	Right lower sacral quadrant	15.18%
	Right upper iliac quadrant	10.71%
	Right lower iliac quadrant	20.54%
Iliac crest/wing	Left Iliac crest/wing	7.59%
	Right Iliac crest/wing	8.48%
Lumbar longitudinal ligament entheses	Lumbar longitudinal ligament entheses	4.91%

### Accuracy of identifying SIJ pathologies among junior readers

During the first round of image review, accuracy of identifying enthesitis was generally low. The accuracy of the three readers was 15.07%, 2.94% and 0.74%, while the accuracy of reporting BME was also less than satisfactory (61.79%, 54.62% and 46.17%). However, the three readers showed moderate to good accuracy regarding erosions (87.53%, 81.53% and 58.42%), fat lesions (86.54%, 77.18% and 87.20%) and ankylosis (80.56%, 89.81% and 91.67%).

Having completed the training sessions on properly reporting enthesitis, the accuracy of the three readers generally improved. For enthesitis, the accuracy improved to 61.03%, 39.34% and 20.22%, which is significantly higher than the first round (*p* < 0.01). Reader 1 and 2 also showed improvements regarding the observations of BME, erosions and fat lesions. Accuracies of the two rounds was reported in Table 4.

**Table 4:** Accuracies of the three readers in identifying the pathologies in the sacroiliac joint MRI images during two rounds of reading exercises using a senior radiologist’s report as gold standard

Accuracy	Reader 1		Reader 2		Reader 3	
	Round 1	Round 2	Round 1	Round 2	Round 1	Round 2
Enthesitis	15.07%	61.03%	2.94%	39.34%	0.74%	20.22%
Bone marrow edema	61.69%	75.05%	54.62%	53.43%	46.17%	31.83%
Erosion	87.53%	92.63%	81.53%	89.90%	58.42%	85.08%
Fatty lesion	86.54%	88.92%	77.18%	88.52%	87.20%	63.85%
Ankylosis	80.56%	75.93%	89.81%	88.89%	91.67%	56.48%

Table 5 presented the Cohen’s kappa (k) of the three readers. It could be seen that the intra-reader agreement of enthesitis was generally poor (0.23, 0.034, 0.014), while BME and erosion were slightly better. Ankylosis showed the highest intra-reader agreement.

**Table 5:** Intra-reader agreement calculated as cohen’s kappa of the three readers in identifying the pathologies of sacroiliac joint MRI

Cohen’s kappa	Reader 1	Reader 2	Reader 3
Enthesitis	0.23	0.034	0.014
Bone marrow edema	0.59	0.48	0.3
Erosion\	0.66	0.36	0.09
Fatty lesion	0.63	0.41	0.2
Ankylosis	0.76	0.79	0.57

## Discussion

This is an accuracy study investigating the accuracy of identifying enthesitis, along with other inflammatory lesions and structural lesions by readers of varying experience, and how the learning progress could have an impact on the readers’ diagnostic performance. Despite the fact that enthesitis is regarded as an inflammatory manifestation in the axial skeleton [13], enthesitis has often taken back seat to other inflammatory lesions, especially BME [14, 15], as well as structural lesions such as erosions and ankylosis, even fat lesions and backfill, which are considered predictors of new bone formation [16], in the evaluation of SIJ MRI. This is partly due to the perception that enthesitis is rarely seen in SIJ MRI, as reported in the earlier reports [17]. Diagnostic trials investigating the diagnostic utility of pelvic enthesitis in the patients suspected of SpA reported that enthesitis had an excellent specificity of over 90%, but the sensitivity was around 20% [10, 17]. However, subsequent studies revealed that enthesitis in the axial skeleton could be more prevalent than previously perceived. One study showed that whole-body MRI identified enthesitis in 78.3% of the axSpA patients, while 33.5% of the enthesitis sites occurred at the pelvis [18]. Agten et al. utilized gadolinium-enhanced fat-saturated T1-weighted (T1 + Gd) sequences to visualize enthesitis at the lumbar spinal ligaments, and showed that interspinous enthesitis and supraspinous enthesitis could be present in 64.7% and 60.3% of the SpA patients [19]. In the SPACE cohort, signs of enthesitis could be found in 68.1% in the entire spine of the early axSpA patients, while the cervical, thoracic and lumbar spine take up 14.3%, 64.8% and 18.7% of the enthesitis lesions, respectively. [20, 21] In the current study, we calculated the prevalence of enthesitis in the SIJ MRI of axSpA patients, reaching 52.23%. The significant discrepancy across the studies regarding the prevalence of enthesitis could be accounted for by the fact that these studies evaluated different areas. One peculiar finding of note is that the current study reported a significantly higher prevalence of enthesitis in axSpA patients than the previous studies with a similar design [10, 17]. It should be noted that both of the older studies were conducted using a 1.5T scanner, as opposed to 3.0T in the current study, which could partly explain the discrepancy, since 3.0T MRI yields approximately 2-fold higher signal-to-noise ratios [22]. It was also possible that the senior radiologist overestimated the prevalence of enthesitis, since the reference was created by only one senior radiologist, who paid extra attention to the enthesitis. The study population is also different from Herregods et al. [10], investigating enthesitis in pediatric patients aged 6–18 years old.

More importantly, we also investigated the association between enthesitis and other imaging findings. We found that in 81.58% of the patients with enthesitis on SIJ MRI, concomitant BME was also detected on SIJ MRI, while 18.42% of the enthesitis occurred independent of BME. This result was in accordance with a previous study, which found that 74% of pelvic enthesitis was accompanied by sacroiliitis [10]. This finding by the current study further substantiated the statement that findings of enthesitis could be complementary to BME in the evaluation of SIJ MRI, serving as additional proof of the existence of axial inflammation. In cases where BME is absent or equivocal in SIJ MRI, enthesitis could assist in the diagnosis of axSpA.

This study also investigated the distribution of enthesitis in SIJ MRI. We divided enthesitis into three areas, namely sacroiliac ligament entheses (excluding interosseous ligament), the longitudinal ligaments of the lumbar vertebrae, and the iliac crest and wing. We found that enthesitis was most prevalent at the posterior sacroiliac ligament entheses inserting into the bilateral lower iliac quadrants, with a prevalence over 20%. This number is significantly higher than that reported by Jans et al. [17], which reported a prevalence of only 2.8%. Whether this higher prevalence in this specific quadrant is merely an overestimation or bona fide awaits further verification. For enthesitis located at the longitudinal lumbar ligaments and iliac crest and wing, the prevalence was around 5% and 8%, respectively, which matched the previous study [17].

An intriguing finding of this study is that enthesitis is very commonly overlooked in the evaluation of SIJ MRI in clinical practice, especially in readers with less experience in musculoskeletal imaging. This study attempted to create an environment simulating the clinical setting, by asking the readers to report the imaging findings of each SIJ MRI scan without knowledge of the objective of this study. The first round of image review confirmed this observation, showing that the accuracy of identifying enthesitis varied from 0.74 to 15.07%, which is far from satisfactory. After revealing the objective of this study and completing a training session and a workshop on the imaging manifestations of the SIJ MRI in axSpA, the three readers presented significantly better accuracy of identifying enthesitis in the second round of image review, albeit still less than satisfactory. The accuracy of observing BME also failed to meet the expectations, ranging between 50 and 60%. On the other hand, the accuracies of erosions, fat lesions and ankylosis were moderate to good. The cohen’s kappa values also showed that the intra-reader agreement was better in the evaluation of structural lesions, as compared with the inflammatory lesions. Ankylosis showed the highest intra-reader agreement, since ankylosis is often unequivocal. This result suggested that for radiologists and rheumatologists with less experience in musculoskeletal imaging, training sessions and workshops are essential so as to achieve higher accuracy in reading exercises, and more attention should be paid to enthesitis. Moreover, how the training sessions and the workshops are executed could bear more significance. In this study, the junior readers were asked to attend both a lecture and an interactive workshop with hands-on exercises reviewing SIJ MRI images of 20 axSpA patients. We believe that teaching and learning is an evolving process which requires the collaboration of the senior readers and the junior readers, and that such knowledge should be reinforced in daily image reviews by means of effective communication between the senior readers and the junior readers. In retrospect, multiple training sessions instead of a single session might further enhance reader calibration.

There were a few issues of note in this study. Different from previous articles, this study excluded the interosseous ligament at the SIJ from the evaluation of enthesitis. This decision was in line with the 2019 updated version of the definitions of SIJ MRI lesions by the ASAS MRI working group [13], arguing that increased signal at the interosseous soft tissues could be difficult to distinguish from vascular signals. One important limitation to this study is that only three readers participated in this reading exercise, and as a consequence the generalizability of this study might be limited. However, the three readers were selected so as to represent readers of varying experience ranging from 1 year to 5 years, and the improved reading accuracies were consistent across the three readers. Including more readers in the future is likely to improve the reliability of the results. This study did not report enthesitis at the pubic symphysis since the FOV adopted in this study could not effectively cover this area. One previous study reported that active inflammatory changes around the pubic symphysis could be seen in 18% of the axSpA patients [23]. Another limitation to this study is that the inflammatory lesions and the structural lesions were reported on a binary scale as opposed to ordinal scale. Significant lesions such as large edema area could hardly be missed even by the less experienced readers. However, grading lesions on a nominal scale could also incur more significant inconsistency between readers.

## Conclusion

In conclusion, junior readers with less experience in musculoskeletal imaging have lower accuracy of identifying inflammatory lesions in the SIJ MRI than the structural lesions, especially enthesitis. Findings of enthesitis could complement bone marrow edema to serve as proof of inflammation in the SIJ. The learning progress could significantly improve the readers’ diagnostic accuracy of enthesitis, along with other inflammatory lesions in the SIJ. It is advised that regular-interval training sessions and workshops should be given to junior readers on how to properly review SIJ MRI scans.

## Data Availability

The datasets used and/or analysed during the current study are available from the corresponding author on reasonable request.

## Abbreviations

MRI: Magnetic resonance imaging
SIJ: Sacroiliac joint
SpA: Spondyloarthritis
axSpA: Axial spondyloarthritis
pSpA: Peripheral spondyloarthritis
ASAS: SpondyloArthritis International Society
BME: Bone marrow edema
T1WI: T1-weighted images
T2-FS: T2-weighted fat-suppressed turbo spin echo
T1-FS: T1-weighted images with fat saturation
STIR: Short time inversion recovery
FOV: Field of view
T1 + Gd: Gadolinium-enhanced fat-saturated T1-weighted
